# Formulation and Evaluation of Topical Nano-Lipid-Based Delivery of Butenafine: In Vitro Characterization and Antifungal Activity

**DOI:** 10.3390/gels8020133

**Published:** 2022-02-18

**Authors:** Ameeduzzafar Zafar, Syed Sarim Imam, Nabil K. Alruwaili, Mohd Yasir, Omar Awad Alsaidan, Sultan Alshehri, Mohammed M. Ghoneim, Mohammad Khalid, Ali Alquraini, Salman S. Alharthi

**Affiliations:** 1 Department of Pharmaceutics, College of Pharmacy, Jouf University, Sakaka 72341, Al-Jouf, Saudi Arabia; nkalruwaili@ju.edu.sa (N.K.A.); osaidan@ju.edu.sa (O.A.A.); 2 Department of Pharmaceutics, College of Pharmacy, King Saud University, Riyadh 11451, Saudi Arabia; salshehri1@ksu.edu.sa; 3 Department of Pharmacy, College of Health Science, Arsi University, Asella 396, Ethiopia; mohdyasir31@gmail.com; 4 Department of Pharmacy Practice, College of Pharmacy, Almaarefa University, Ad Diriyah 13713, Saudi Arabia; mghoneim@mcst.edu.sa; 5 Department of Pharmacognosy, College of Pharmacy, Prince Sattam Bin Abdulaziz University, P.O. Box 173, Al-Kharj 11942, Saudi Arabia; drkhalid8811@gmail.com; 6 Department of Pharmaceutical Chemistry, Faculty of Clinical Pharmacy, Al Baha University, Al Baha 65779, Saudi Arabia; aalquraini@bu.edu.sa; 7 Department of Pharmacy, Security Forces Hospital Program, Riyadh 11481, Saudi Arabia; salharthi@sfh.med.sa

**Keywords:** butenafine, bilosomes, permeation, irritation study, antifungal activity

## Abstract

The present research work was designed to prepare butenafine (BN)-loaded bilosomes (BSs) by the thin-film hydration method. BN is a sparingly water-soluble drug having low permeability and bioavailability. BSs are lipid-based nanovesicles used to entrap water-insoluble drugs for enhanced permeation across the skin. BSs were prepared by the thin-film hydration method and optimized by the Box–Behnken design (BBD) using lipid (A), span 60 (B), and sodium deoxycholate (C) as independent variables. The selected formulation (BN-BSo) was converted into the gel using Carbopol 940 as a gelling agent. The prepared optimized gel (BN-BS-og) was further evaluated for the gel characterization, drug release, drug permeation, irritation, and anti-fungal study. The optimized bilosomes (BN-BSo) showed a mean vesicle size of 215 ± 6.5 nm and an entrapment efficiency of 89.2 ± 1.5%. The DSC study showed that BN was completely encapsulated in the BS lipid matrix. BN-BSog showed good viscosity, consistency, spreadability, and pH. A significantly (*p* < 0.05) high release (81.09 ± 4.01%) was achieved from BN-BSo compared to BN-BSog (65.85 ± 4.87%) and pure BN (17.54 ± 1.37 %). The permeation study results revealed that BN-BSo, BN-BSog, and pure BN exhibited 56.2 ± 2.7%, 39.2 ± 2.9%, and 16.6 ± 2.3%. The enhancement ratio of permeation flux was found to be 1.4-fold and 3.4-fold for the BN-BS-og and pure BN dispersion. The HET-CAM study showed that BN-BSog was found to be nonirritant as the score was found within the limit. The antifungal study revealed a significant (*p* < 0.05) enhanced antifungal activity against *C. albicans* and *A. niger*. The findings of the study revealed that BS is an important drug delivery system for transdermal delivery.

## 1. Introduction 

The transdermal delivery system has been of great interest to researchers, considering the low permeability of the stratum corneum, which represents the main barrier for the skin absorption of exogenous substances [1]. It is an alternative delivery route for drug administration. It has a wide range of advantages over the oral route such as preventing the first-pass metabolism and avoiding drug degradation [2]. Different vesicular systems have been used to enhance the drug permeation by altering the stratum corneum, i.e., losing the intercellular lipid barriers.

The ingredients used in the vesicular system play an important role in the drug permeation by opening the tight junction of the SC [3]. Different lipid-based delivery systems, such as bilosomes [4], ethosomes [5] niosomes [6], transferosomes [7], and liposomes [8] have been used to enhance the permeation and absorption of poorly soluble drugs. Among them, bilosomes (BSs) are novel vesicular drug carriers containing bile salt [4]. The use of negatively charged bile salt (sodium-deoxycholate) enhances the stability of BS as well as permeation through the SC of the skin by the fluidizing effect [9]. Different bilosomes have been prepared and evaluated for in vitro and in vivo studies [10,11,12]. They have reported nano-sized vesicles and enhanced permeation and release of tested drugs. 

The administration of antifungal drugs using different vesicular deliveries by the transdermal route has been reported for drugs such as fluconazole [13] ketoconazole [14], itraconazole [15], ketoconazole [16], and clove oil [17] to avoid the limitation with the oral delivery. Butenafine (BN) is a broad-spectrum antifungal drug that belongs to benzylamine and has a potent fungicidal activity [18]. It acts by preventing the biosynthesis of ergosterol and inhibiting the squalene monooxygenase enzyme [19]. It is slightly soluble in water and readily soluble in organic solvents. There are different BN-loaded nanoformulations: PLGA-NP-laden chitosan nano-gel and NLCs-based gel were prepared and evaluated for different parameters [20,21]. They have reported prolonged drug release and enhanced drug permeation and retention with antifungal activity. In another study, Ahmed et al. prepared and evaluated a butenafine nanosponge-based gel by the solvent emulsification method [19]. The prepared formulation exhibited an enhanced flux of 0.18 (mg/cm^2^ h) and a drug diffusion of 89.90 ± 0.87% in 24 h. It also exhibited effective activity against pathogenic fungal strains.

The objective of the present research work was to develop BN-loaded bilosomes (BN-BS) and optimize them using the Box–Behnken design. The optimized bilosomes (BN-BSo) were further incorporated into the Carbopol to increase the therapeutic activity. The optimized bilosomes gel (BN-BSog) was further evaluated for in vitro release, in vitro permeation, irritation study, and antifungal study.

## 2. Result and Discussion

### 2.1. Optimization

**Figure 1:**
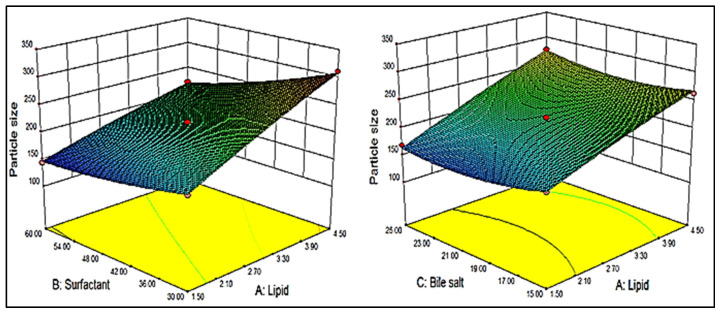
3D graph depicting the effect of lipid, surfactant and bile salt on vesicle size (Y_1_).

**Figure 2:**
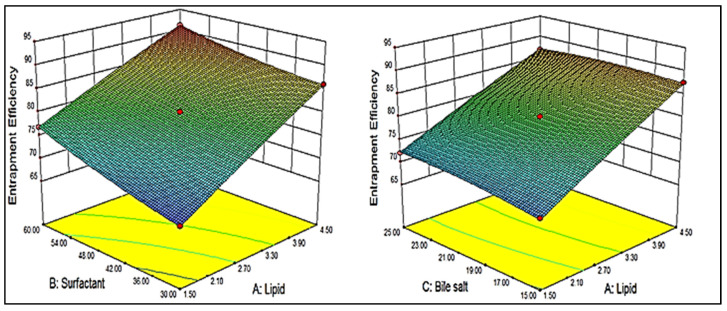
3D graph depicting the effect of lipid, surfactant and bile salt on entrapment efficiency (Y_2_).

**Figure 3:**
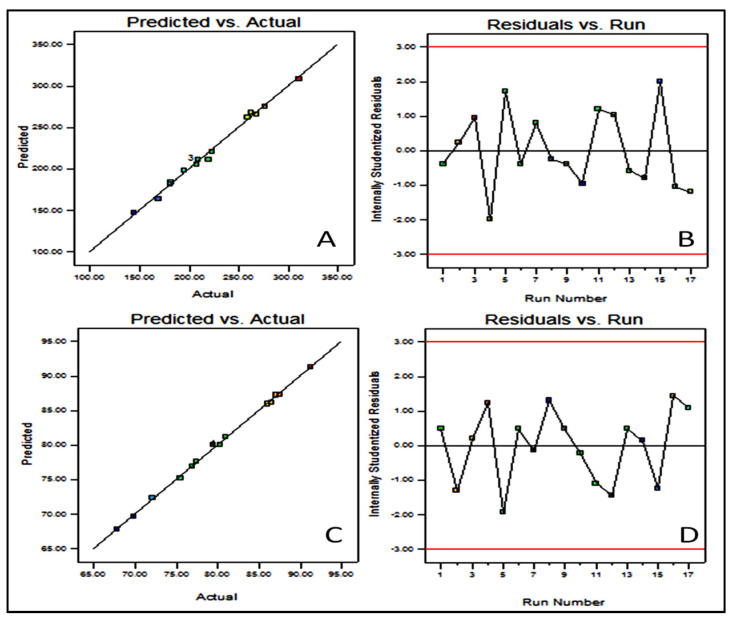
(**A**) Actual and predicted value graph of vesicle size; (**B**) residual vs. experimental run of vesicle size; (**C**) actual and predicted value graph of entrapment efficiency; (**D**) residual vs. experimental run of entrapment efficiency.

BBD showed 17 different formulation compositions with 5 common formulas (center point), as depicted in Table 1. The prepared formulations particle size (Y_1_) and entrapment efficiency (Y_2_) were determined. These experimental values were added to the BBD software to evaluate the other statistical parameters. The predicted response was designated by the second-order polynomial equations. The suitability of BBD was expressed by a high value of the coefficient of determinants (0.9932 Y_1_ and 0.9985 Y_2_). The high value of the regression coefficient and a significant (*p* < 0.05) effect of the independent variable was observed on the dependent variables [22]. A three-dimensional graph was constructed for each response showing the effect of lipid, surfactant, and bile salt over the vesicle size (Y_1_, Figure 1) and entrapment efficiency (Y_2_, Figure 2). The experimental and predicted value plot was also constructed (Figure 3), which explain the closeness between the actual and predicted value [23]. 

### 2.2. Effect of Independent Variables on Vesicle Size (Y_1_)

The design showed seventeen formulation compositions, and a significant (*p* < 0.05) variation in size was observed by changing the ratio of independent variables. The formulation (F3) prepared with lipid (1.5%), surfactant (60 mg), and bile salt (20 mg) showed the lowest mean vesicle size (144.4 ± 4.8 nm). The formulation (F2) prepared with lipid (4.5%), surfactant (30 mg), and bile salt (20 mg) showed the maximum mean vesicle size (310.1 ± 7.4 nm). Each independent variable showed an effect on the size, as depicted in Figure 1. At high or low levels of independent variables, positive and negative effects on the size were exhibited. At a fixed concentration of surfactant (B) and bile salt (C), the vesicle size increased with lipid concentration (A). This might be due to the presence of excess lipid being responsible for the enhancement of viscosity of the dispersion and thickness of the bilayer [24]. Moreover, there was a high chance of incomplete emulsification (due to lack of surfactant), which was responsible for the aggregation of BS. The surfactant (B, span 60) played a negative effect on vesicle size. With increasing surfactant concentration, the vesicle size decreased, which might be due to the reduction in surface energy between the aqueous and lipid phase, and these findings are supported by the previously reported literature [25,26]. Bile salt (C, SD) exhibited a negative effect on vesicle size, and as the concentration of SD increased, the vesicle size of BS decreased. This may be due to the surfactant property of bile salt (SD), which helps to reduce the interfacial tension, as well as enhance the stability of BS.

The overall effect of independent variables on particle size (Y_1_) can be represented by the obtained polynomial, Equation (1):

Particle size (Y_1_) = + 211.33 + 49.62A − 31.08B − 2.57C − 12.76AB + 6.37AC − 0.88BC − 3.41A^2^ + 7.00B^2^ + 14.26C^2^ (1)

The positive and negative signs of the above equation show the positive and negative effects on the vesicle size of BS. The lipid (A) showed a positive effect and span 60 (B) and bile salt (C) showed a negative effect. Here, A, B, AB, AC, B^2^, and C^2^ were significant model terms, i.e., these terms showed a significant (*p* < 0.05) effect on vesicle size in a positive or negative direction, while terms C, BC, and A^2^ exhibited an insignificant effect. In this case, the best fit model was found to be quadratic, which could be justified by the higher value of the regression coefficient (R^2^ = 0.9932), as shown in Table 2. The desired F value (F = 114) and the lack of fit were found to be insignificant with the *p* = 0.2818 (*p* > 0.05). The regression values of all models and results of ANOVA of the best-fitted model (quadratic) represented in Table 2 and Table 3. 

### 2.3. Effect of Independent Variables on Entrapment Efficiency (Y_2_)

BBD depicted seventeen formulation compositions with five center points (common composition to check the error). A significant (*p* < 0.05) variation in the entrapment efficiency (Y_2_) was observed by changing the composition of lipid, surfactant, and bile salt. The formulation (F1) prepared with lipid (1.5%), surfactant (30 mg), and bile salt (20 mg) showed the lowest mean entrapment efficiency (67.79 ± 1.5%). The formulation (F2) prepared with lipid (4.5%), surfactant (60 mg), and bile salt (20 mg) showed the maximum mean entrapment efficiency (91.29 ± 2.1%). Each independent variable showed an effect on the entrapment efficiency, as depicted in Figure 2. The high or low levels of independent variables showed a negative or positive effect on the entrapment efficiency. As shown in Figure 2, the lipid concentration (A) at a fixed concentration of surfactant (B) and bile salt (C), increased the entrapment efficiency increases. This finding may be due to the availability of excess space to accommodate BN. It helps to enhance the stability, as well as to prevent the leakage of BN from the lipid bilayer [27]. The surfactant showed a dual effect on the entrapment efficiency. The initial increase in surfactant concentration increased the entrapment and, after the intermediate level of surfactant, the entrapment of BN decreased. The entrapment of BN increased until sufficient lipid was available to accommodate BN due to the increased solubility. The longer alkyl chain length and low HLB of span 60 led to a higher entrapment efficiency [28]. Bile salt (sodium deoxycholate, SD) exhibited a positive effect on entrapment efficiency. The surface-active property, as well as ionic nature (increases the repulsion), helped to increase the entrapment efficiency. The bile salt incorporated in the lipid bilayer membrane, which enhances the lipid bilayer flexibility as well as the internal core structure, also increased, leading to a high BN encapsulation [29]. Further, it also exhibited a positive impact on the drug lipid solubility due to the surface-active property and, hence, increased the entrapment efficiency [30].

The overall effect of independent variables on entrapment efficiency (Y_2_) can be represented by the obtained polynomial, Equation (2):

Entrapment efficiency (Y_2_) = + 80.07 + 8.15A + 3.63B + 0.66 C − 0.95 AB − 0.71 AC + 1.86 BC − 0.26A^2^ + 0.68 B^2^ − 0.67 C^2^ (2)

The positive and negative signs in the polynomial equation showed the synergistic and antagonistic effect over the entrapment efficiency. The factors A, B, C, AB, AC, BC, B^2^, and C^2^ were found as significant model terms (*p* < 0.05), i.e., these terms showed a significant effect on entrapment efficiency. The other term A^2^ exhibited an insignificant effect on the entrapment efficiency. In this case, the best-fitted model was quadratic, which could be justified by the higher value of the regression coefficient (R^2^ = 0.9985) and desired F value (F = 521.14) (Table 2). The lack of fit was found to be insignificant with a *p*-value of 0.4221 (*p* < 0.05). The statistical regression values and ANOVA result of the best fit quadratic model is shown in Table 3**.**

### 2.4. Point Prediction

Based on the desired vesicle size and entrapment efficiency, the BN-BSo prepared with lipid (3.5%), surfactant (45 mg), and SD (22 mg) showed a vesicle size of 215 ± 6.5 nm and entrapment efficiency of 89.2 ± 1.5%. The software showed the predicted vesicle size and entrapment efficiency of 221.8 nm and 82.8%, respectively, with a prediction error of <0.5%. The overall desirability value was found to be closer to unity (0.994). A close agreement in the actual and predicted vesicle size and entrapment efficiency was observed (Figure 3A–D). From the results, we can say that the used model and method was found to be robust and reproducible.

### 2.5. Vesicle Size, PDI, and Zeta Potential

**Figure 4:**
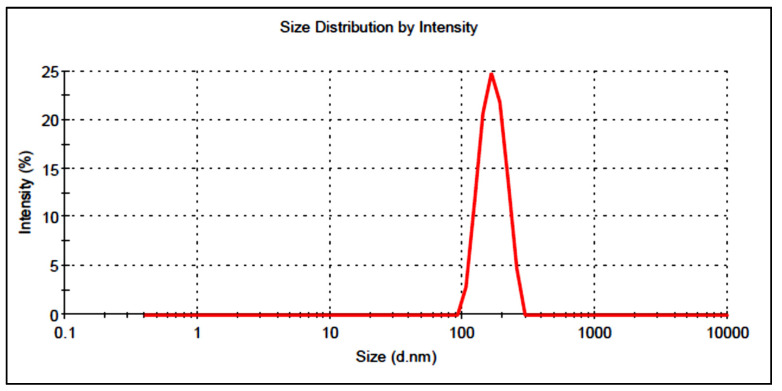
Vesicle size of optimized butenafine bilosomes (BN-BSo) using zeta sizer.

The vesicle size of the prepared BN-BS formulations was found in the range of 144.43 ± 4.8 (F3) to 310.92 ± 7.4 (F2). The optimized composition (BN-BSo) showed a vesicle size value of 215 ± 6.5 nm (Figure 4). PDI was found to be 0.35, which is under the standard limit <0.5, revealing a homogeneous distribution. The zeta potential was found to be highly negative (−45 mV), indicating that the prepared BN-BSo was highly stable and the vesicles were not coagulated/aggregated.

### 2.6. Entrapment Efficiency

The entrapment efficiency of prepared BN-BS formulations was found in the range of 67.79 ± 1.5% (F1) to 91.29 ± 2.1% (F4). The optimized formulation BN-BSo showed the entrapment of 89.2 ± 1.5%. 

### 2.7. Thermal Analysis

**Figure 5:**
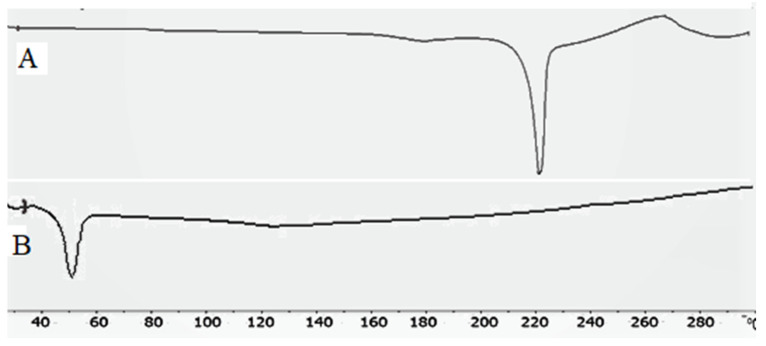
Thermal spectra of (**A**) butenafine and (**B**) BN-BSo.

Figure 5A,B shows the thermal image of BN and BN-BSo. The BN exhibited the characteristic endothermic peaks at 220.5 °C (Figure 5A), indicating its corresponding melting point. The prepared formulation BN-BSo shown did not show any peak of BN in the BN-BSo at their respective melting point (Figure 5B). The absence of the drug peak in BLs was due to the complete entrapment into the lipid bilayer or the solubilization of the drug in the lipid matrix. A similar type of finding was reported in the literature in a BN nanosponge [19] and tenoxicam bilosomes [2].

### 2.8. Development of BN-BSo-Gel

The BN-BSo formulation was successfully incorporated into the gel using Carbopol 940 (1%) as a gelling agent. The selection of the optimum gelling agent was performed by preparing the gel at three different concentrations of Carbopol (0.75%, 1%, and 1.25%). The gel prepared at a Carbopol concentration of (1% *w*/*v*) showed the optimum viscosity, spreadability, and extrudability. Thus, the BN-BSo prepared with lipid (3.5%), surfactant (45 mg), and SD (22 mg) were used to prepare transdermal gel using the Carbopol concentration (1% *w*/*v*).

### 2.9. Viscosity, pH, and Drug Content

BN-BSog was evaluated for viscosity, pH, and drug content. The formulation showed a viscosity of 1322 ± 14 cps, which has been reported for good retention over the skin. The pH was found to be 6.3 ± 0.2, which is compatible with skin pH. BN-BSog exhibited a drug content of 99.2 ± 3.12%. From the result, the maximum amount of drug present in the gel can be found with a uniform distribution of BN. The spreadability value was expressed in the area and it is a very important parameter for the spreading of gel over the skin. The result showed a 5.84 ± 0.24 cm^2^ area, designated as a respectable spreading capacity.

### 2.10. In Vitro Release Study

**Figure 6:**
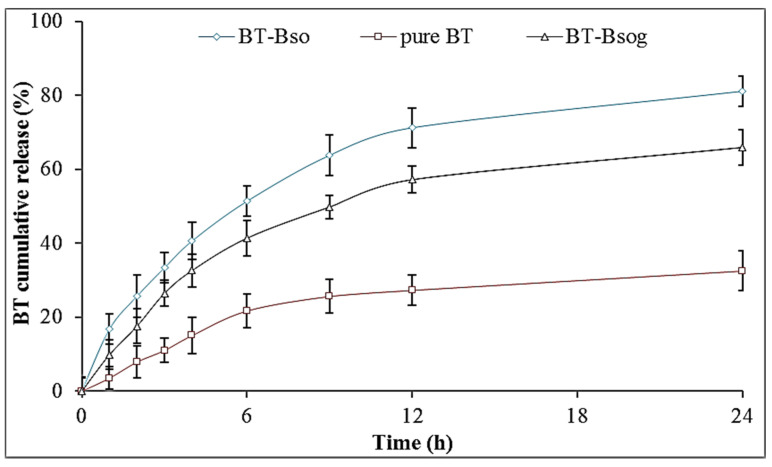
Comparative in vitro release profile of optimized butenafine bilosomes (BN-BSo), butenafine bilosomes gel (BN-BSog), and pure butenafine (BN) using dialysis bag. Study performed in triplicate and data shown as mean ± SD.

The release study was performed to evaluate the release behavior from different prepared formulations (BN-BSo, BN-BSog, and pure BN), and the results are expressed graphically in Figure 6. BN-BSo, BN-BSog, and conventional BN-gel showed the maximum BN release of 81.09 ± 4.01%, 65.85 ± 4.87%, and 17.54 ± 5.37% in 24 h, respectively. The pure BN showed a poor release due to the poor water solubility. After the encapsulation of BN into bilosomes, the release was found to be significantly high (*p* < 0.05). The presence of surfactant and nanometric size led to an enhanced drug release. The smaller-sized particle had a greater effective surface area for absorption. The surfactant and bile salt helped to reduce the surface interfacial tension, which increased the solubility and dissolution of BN in release media. A fast release of BN was found in the initial 1 h due to the presence of BN on the surface of BS, and a later sustained release was found due to slow diffusion from the lipid matrix [12,31]. There was a slower BN release from the prepared BN-BSog formulation than BN-BSo. The presence of an additional barrier of a Carbopol matrix gave a slower and prolonged drug release. The drug particle needs to cross the two layers to reach the release media. A slower drug release is ideal for greater drug absorption. The slow-release profile from BN-BSog is helpful for the transdermal delivery because an initial fast release improves the BN permeation and the later slow and sustained release helps to maintain the therapeutic concentration for a prolonged time [32]. The release data fitted to various release kinetic models and the data showed the Korsmeyer–Peppas model as the best release kinetic model because it had a maximum regression value (R^2^ = 0.9663). The release exponent *n* = 0.45 (0.43 < *n* < 0.85) indicated that the release mechanism was anomalous transport.

### 2.11. In Vitro Permeation Study

**Figure 7:**
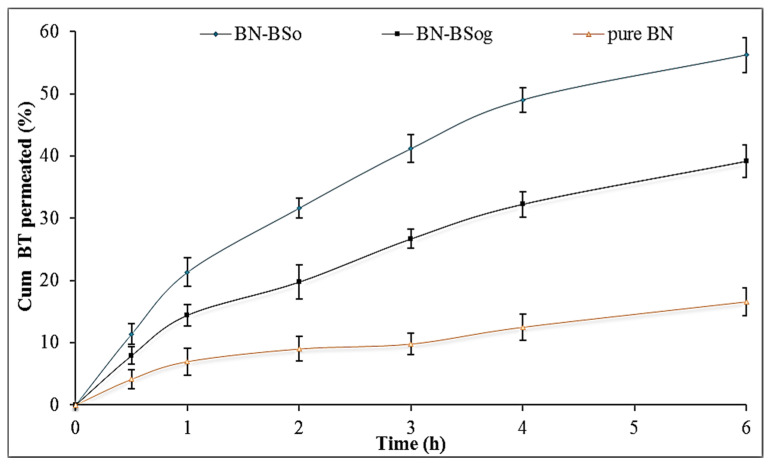
Comparative permeation profile of butenafine bilosomes (BN-BSo), butenafine bilosomes gel (BN-BSog), and pure butenafine (BN) using egg membrane. Study performed in triplicate and data shown as mean ± SD.

Figure 7 illustrates the permeation profile of pure BN, BN-BSo, and BN-BSog across the egg membrane. The amount of BN permeation (%) was found to be 56.2 ± 2.7% from BN-BSo, 39.2 ± 2.9% from BN-BSog, and 16.6 ± 2.3% from pure BN. The flux was also calculated from the prepared formulations. BN-BSog showed a flux value of 10.35 µg/h/cm^2^ and was found to be significantly (*p* < 0.05) lesser than BN-BSo (14.87 µg/h/cm^2^). A higher flux was found from the prepared bilosomes due to the lower viscosity and quicker release of BN in the presence of surfactant. The surfactant and bile salt help to permeate the drug by opening the tight junction of the membrane. The pure BN showed a lower flux (4.38 µg/h/cm^2^) value due to the poor solubility and permeability. The APC was also calculated and the result showed that BN-BSo, BN-BSog, and pure BN dispersion exhibited 11.81 × 10^−3^, 8.21 × 10^−3^, and 3.48 × 10^−3^, respectively. The enhancement ratio between each sample was calculated, and BN-BSo showed 1.4-fold and 3.4-fold increases compared to BN-BSog and pure BN dispersion. BN-BSog exhibited a significantly high flux (2.36-fold) than pure BN dispersion.

### 2.12. Irritation Study

The irritation study of the negative control (0.9% *w*/*v* NaCl), positive control (1% *w*/*v* sodium lauryl sulphate), and prepared BN-BSog was evaluated using the HET-CAM test. The score from all treated groups was evaluated at different time points and the mean irritation score is given in Table 4. The negative control sample at each time point showed a zero score (no irritation, 0–0.9), and the positive control showed a very high score (17.44). The sample showed a score between 9 and 21, considered as highly irritant. The prepared BN-BSog also showed a score of zero, indicating no irritation over the CAM. The zero scores of negative control and the prepared formulation were found to be nonirritant to the membrane, so it was considered as nonirritant to the skin. 

### 2.13. Antifungal Study

**Figure 8:**
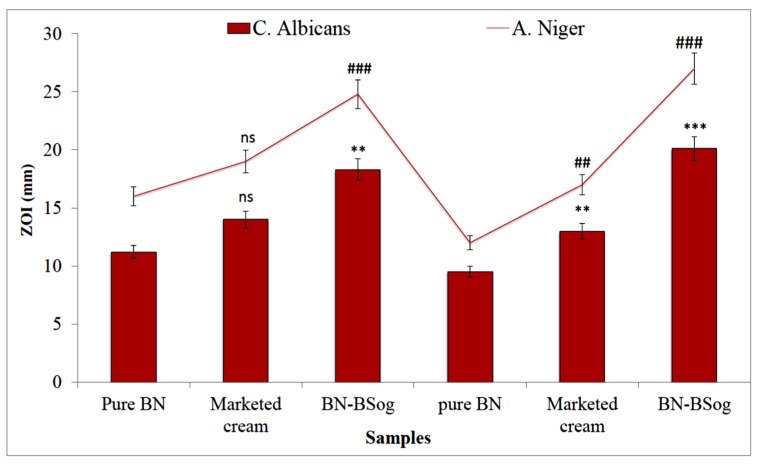
Comparative antifungal activity of butenafine bilosomes gel (BN-BSog), marketed cream, and pure BN against *C. albicans* and *A. niger.* Study performed in triplicate and data shown as mean ± SD. Dunnett’s multiple comparison test was performed between each group, and pure BN was considered as control. ns: nonsignificant; **, ## significant to pure BN (*p* ˂ 0.01); and ***, ### highly significant to pure BN (*p* ˂ 0.001).

The anti-fungal activity of the pure BN, marketed cream, and BN-BSog was determined against two different fungal strains (*C. albicans and A. niger*). The ZOI of the treated samples was evaluated, and the result was noted at 24 h and 48 h (Figure 8). The pure BN, marketed cream, and BN-BSog showed that the ZOIs against *C. Albicans* were 11.2 ± 1.3 mm, 14 ± 0.8 mm, and 18.3 ± 2.1 mm at 24 h, respectively. A significant (*p* ˂ 0.001) increase in ZOI was achieved at 48 h from BN-BSog (20.1 ± 1.5 mm). The pure BN and marketed cream showed a lower activity at 48 h and the ZOI was slightly decreased. Similarly, the samples (pure BN, marketed cream, and BN-BSog) were treated with *A. niger*, and the ZOI was found to be 16 ± 1.4 mm, 19 ± 1.6 mm, and 24.8 ± 1.9 mm at 24 h, respectively. The plates were kept for another 24 h and the result was noted. The results showed a significant (*p* ˂ 0.01) reduction in ZOI from pure BN (12 ± 0.7) and a slight reduction from the marketed cream (17 ± 2.1 mm). The prepared BN-BSog showed a significant (*p* ˂ 0.001) enhancement in the ZOI (27 ± 1.7 mm). A higher activity was achieved from BN-BSog due to the greater solubility of BN in the bilosomes. The presence of surfactant and bile salt helped to solubilize the BN and also reduce the size. The smaller-sized particles had the greater effective surface area, leading to higher BN absorption. The data showed that BN was more active against *A. niger* than *C. albicans*. As per antimicrobial classification activity, ZOI > 20 mm indicated strong activity and the ZOI of BN-BSog showed a ZOI of >20 mm against both strains, so it revealed that the prepared BN-BSog had a strong antifungal activity.

## 3. Conclusions

The present research work was designed to prepare a BN-BS carrier for transdermal delivery for antifungal activity. BN-BS was successfully developed by the thin-film hydration method and further optimized by the Box–Behnken design. The prepared BN-BS exhibited nanometric-size vesicles with greater entrapment efficiency. The optimized composition of BN-BS with desirable effect was successfully transformed in gel using Carbopol 940 as a gelling agent. The prepared BN-BSog exhibited optimum viscosity, spreadability, consistency, and compatible pH. It showed a significantly high BN release and permeation compared to pure BN dispersion. The formulation was tested for irritation potential using the HET-CAM test score, which revealed it to be nonirritant, similar to the negative control. No damage to the blood capillaries was observed. BN-BSog exhibited a higher antifungal activity than pure BN and marketed cream. Our findings explored the bilosomes as a drug carrier for transdermal delivery.

## 4. Materials and Experimental

### 4.1. Materials

Butenafine was received as a gift sample from Jazeera Pharmaceutical Industries (Riyadh, Saudi Arabia). The lipid (phosphatidyl choline, CAS: 8002-43-5), bile salt (sodium deoxycholate, CAS: 302-95-4), and dialysis bag (MW cutoff 12–14 kDa) were procured from Sigma Aldrich (St. Louis, MO, USA). Cholesterol (CAS: 57-88-5) was purchased from Alpha Chemika, Mumbai, India. The solvents Methanol CAS: (67-66-1) and chloroform (CAS: 67-66-3) were procured from Fisher Scientific, Loughborough, LE11 5RG, UK. All other chemicals used in this research work were of analytical grade.

### 4.2. Experimental 

#### 4.2.1. Preliminary Study

A preliminary study was performed to select the lower and upper levels of the independent variables. The different formulations were prepared with lipid, span 60, SD, and CH. The prepared formulations were evaluated for size, PDI, and encapsulation efficiency. The study started with the lowest concentration for the formation of bilosomes and then kept for 24 h to evaluate the formation of a uniform phase. At very low concentrations, the formation of a uniform phase did not take place. The concentration of ingredients was gradually increased to check the formation of bilosomes with nano-size, low PDI, and optimum encapsulation. At very high concentrations, large-size bilosomes formed with greater PDI values. Thus, the level of independent variables lipid (1.5 to 4.5%), surfactant (30 mg to 60 mg), and bile salt (15 mg to 25 mg) were selected for the formulation of bilosomes. The methanol and chloroform blend was selected as the organic solvent because it showed the maximum solubility of ingredients. 

#### 4.2.2. Optimization

After the preliminary screening study, the optimization of BS was performed using the Box–Behnken design (Design expert software, Statease, Minneapolis, Minnesota, United States). The design displayed replicating center points and a set of points designated at the mid edge of the multidimensional cube. It was used to evaluate the main effects, interaction effects, and quadratic effects of the formulation ingredients [33]. In this research study, BBD was used to optimize the formulation, and the design depicted 17 formulation runs. From the preliminary study, the independent constraints lipid (1.5% as low to 4.5% as high), surfactant (30 mg as low to 60 mg as high), and bile salt (15 mg as low to 25 mg as high) were selected. Their influences on particle size (Y_1_, nm) and entrapment efficiency (Y_2_, %) as dependent variables were investigated, as shown in Table 5. The numerical data of particle size and entrapment efficiency were fitted into various formulation design models, and the regression analysis, as well as analysis of variance, was calculated to determine the optimized model. Three-dimensional surface response graphs, actual and predicted with residual plots, were also constructed to interpret the results. The general model quadratic equation (3) obtained from the BBD can be represented as

Response (Y) = β_0_ + β_1_A + β_2_B + β_3_C + β_12_AB + β_13_AC + β_23_BC + β_11_A^2^ + β_22_B^2^ + β_33_C^2^ (3)

where Y is the measured response of each factor; A, B, and C are the values of independent constraints; β_0_ is a constant; β_1_, β_2_, and β_3_ are the linear coefficients; β_12_, β_13_, and β_23_ are the interaction coefficients; and β_11_, β_22_, and β_33_ are the quadratic coefficients of the experimental values.

#### 4.2.3. Formulation of Bilosomes

Bilosomes were prepared by the thin-film hydration method [10] and the composition is shown in Table 1. BSs were prepared with BN, lipid, span 60, SD, and a fixed amount of CH (25 mg). The weighed quantity of ingredients was dissolved in the chloroform-methanol mixture (10 mL) with stirring and sonication. The organic solution was transferred to a round-bottom flask and fixed to the rotary evaporator (BUCHI, Switzerland). The organic solvent was evaporated at 40 °C at reduced pressure (150 mbar). The flask was rotated at 100 rpm until the complete organic solvent was evaporated. The thin film was formed on the wall of the flask and kept in a desiccator for 24 h for complete removal of the organic solvent. Finally, the lipid film was dehydrated using phosphate buffer (10 mL) in a rotatory evaporator for 60 min. The prepared BN-BS were collected, kept overnight at 4 °C, and then further sonicated for 3 cycles for 1 min at an interval of 5 min. BN-BS were stored in the refrigerator for further characterization. 

### 4.3. Characterization of BN-BS

#### 4.3.1. Particle Characterization

The double-distilled water-diluted samples of BN-BS were placed into a quartz cuvette and then analyzed for the vesicle size and PDI by a zeta sizer (Zetasizer Nano, Malvern, UK). The same sample was analyzed for the determination of zeta potential by using a specific cuvette containing an electrode.

#### 4.3.2. Entrapment Efficiency

The entrapment efficiency of BN-BS formulations was determined by the indirect method using the ultracentrifugation technique. The samples were filled into a centrifugation tube and then centrifuged at a speed of 10,000 rpm using a cooling centrifuge (Eppendorf, Centrifuge, Hamburg, Germany). The supernatant was separated and the absorbance was analyzed by a UV-spectrophotometer at 251 nm. The % EE was calculated by the following equation:

(4) $$\%\;\mathrm{EE}=\frac{\mathrm{Total}\;\mathrm{BN}-\mathrm{BN}\;\mathrm{in}\;\mathrm{supernatant}}{\mathrm{Total}\;\mathrm{BN}}\times 100$$

#### 4.3.3. Thermal Analysis

The thermal spectra of BN and the optimized formulation (BN-BSo) were analyzed by differential scanning calorimetry (Mettler Toledo, USA). Each sample (approximately 3 mg) was taken and packed into an aluminium pan. The pan was placed into a calorimeter with the blank aluminium pan as a reference. The samples were scanned under a nitrogen inert environment at 20–400 °C and spectra were captured.

#### 4.3.4. Development of BN-BSs Loaded Gel

The optimized bilosomes (BN-BSo) were loaded into a suitable gelling agent for longer retention over the epidermis. The optimized butenafine-loaded bilosomes gel (BN-BSog) was prepared by using carbopol 940 (1% *w*/*v*) as a gelling agent. The overnight-swelled carbopol 940 was added into BN-BS dispersion and BN dispersion. The samples were kept overnight for complete swelling and gelling of the polymer [34]. The gels were added with triethanolamine (0.5%) and methylparaben (0.1%) to adjust the pH and store them for further evaluation.

#### 4.3.5. Gel Characterization

The viscosity of the prepared gel was evaluated on a Brook field viscometer (V420001, Fungi Lab, Sant Feliu Llobregat, Spain). The study was performed by using the spindle C-50-1 at room temperature. The pH of BN-BSog was measured by a digital pH meter (Hi2211, Hanna, Europe) by keeping the diluted sample pH meter. The sample pH was measured by dipping the electrode for 2 min, and then the pH value was noted. The BN content in the gel was determined by taking BN-BSog (1 g) and dissolving in methanol (10 mL). The sample was sonicated in a bath sonicator and then analyzed by a UV-spectrophotometer. The spreadability of the BN-BSog was analyzed by placing it on a clean Petri dish, and the initial diameter was noted. The second petriplate was placed over the first petriplate and a weight (100 g) was placed for 30 s. The final diameter was noted and the spreadability percentage was calculated by the given equation:

(5) $$\mathrm{Spreadability}\;\left(\%\right)=\frac{\mathrm{Final}\;\mathrm{diameter}-\mathrm{Initial}\;\mathrm{diameter}}{\mathrm{Initial}\;\mathrm{diameter}}\times 100$$

#### 4.3.6. In Vitro Release Study

The release study was performed by using a pretreated dialysis bag in the phosphate buffer with ethanol (70:30) as release media (250 mL). The temperature was fixed at 32 ± 0.5 °C with a stirring speed of 50 rpm. The samples of BN-BSo, BN-BSog, and BN-gel (equivalent to 2.5 mg of BN) were placed into a dialysis bag and immersed in the release media. The released content (2 mL) was taken at a definite time interval (1, 2, 3, 4, 6, 9, 12, 24 h) and replaced with fresh release media. The concentration was analyzed by a UV spectrophotometer at 251 nm in triplicate. The cumulative drug release (%) was calculated and the graph was plotted between % cumulative drug release vs. time. The release data of BN-BSog were fitted into kinetic models such as the zero-order, first-order, Higuchi [35], and Korsmeyer–Peppas model. The graph was plotted and the R^2^ value was calculated to select the best-fit kinetic release model.

#### 4.3.7. Permeation Study

The permeation study was performed by using the egg membrane due to the resemblance in the human skin stratum corneum [36,37]. The eggs were collected from the local poultry farm and kept in dilute hydrochloric acid solution (0.1 N) for 2 h. The eggshell was dissolved and the membrane was separated. The membrane was rinsed with deionized water and dried at room temperature. The membrane was placed between the donor and acceptor compartment of the diffusion cell (volume 22 mL, area 1.2 cm^2^). The permeation media phosphate buffer with ethanol (pH 6.8, 10 mL) was filled into the acceptor compartment (stirring speed 50 rpm) and the temperature was fixed at 32 ± 0.5 °C. The samples BN-BSo, BN-BSog, and BN-gel (equivalent to 2 mg BN) was filled into the donor compartment. At a definite time (0.5, 1, 2, 3, 4, and 6 h), released aliquots (1 mL) from the acceptor compartment were collected and replaced with the same volume of blank permeation media. The aliquots were filtered with a membrane filter (0.25 µm) and the concentration was analyzed by a UV-vis spectrophotometer at 251 nm. The amounts of BN permeated, flux, permeation coefficient (PC), and enhancement ratio (ER) were calculated.

(6) $$\mathrm{PC}\;=\frac{\mathrm{Flux}}{\mathrm{Area}\;\mathrm{of}\;\mathrm{membrane}\;\times \mathrm{Initial}\;\mathrm{BN}\;\mathrm{amount}}$$

(7) $$\mathrm{ER}=\frac{\mathrm{PC}\;\mathrm{of}\;\mathrm{BN}-\mathrm{BSog}}{\mathrm{PC}\;\mathrm{of}\;\mathrm{the}\;\mathrm{conventional}\;\mathrm{BN}\;\mathrm{gel}}$$

#### 4.3.8. Irritation Study

The irritation of the BN-BSog was evaluated by using an in vitro HET-CAM test, which is an alternative to the skin irritation test. This test was used because the blood vessel of the CAM is similar to those of humans and other test animals [38]. The fertilized hen eggs were procured from the local poultry farm and incubated for 10 days in an incubator at 37 ± 0.5 °C/60 ± 1% RH. On the 10_th_ day, the egg was taken out from the incubator and the shell above the air chamber was removed. The inner membrane appeared, which was directly in contact with the CAM. It was moistened with NaCl solution (0.9%) and the membrane was carefully removed using forceps. The samples BN-BSog, sodium lauryl sulphate (1% *w*/*v*, positive control), and 0.9% NaCl (negative control) were added to the CAM and the score was noted for different time points up to 5 min. The score was given according to the scale as no irritation (0–0.9), mild irritation (1–4.9, mild decolorization), moderate irritation (membrane decolorization, 5–8.9), and full hemorrhage (severe irritant, 9–21).

#### 4.3.9. Antifungal Study

The antifungal activity of BN-BSog, pure BN, and marketed cream (Mentax cream 1%, Mylan Pharmaceuticals Inc. San Carlos, CA, USA.) was determined in fungal strains (*C. albicans* and *A. niger*) by using the cup plate method. The nutrient agar medium (Sabouraud dextrose agar) was prepared and sterilized at 121 °C for 15 min using an autoclave (CABN60801, Astell, England). The microorganism’s culture (0.1 mL) was mixed with sterilized melted Sabouraud dextrose agar (10 mL) and transferred into a sterilized disposable plastic petriplate under aseptic conditions. After complete solidification, 6 mm of the well was made using a sterilized borer. Each sample of BN-BSog, pure BN, and marketed cream was filled into the cup and maintained for 1 h at room temperature. The plate was placed into an incubator (Binder, Camarillo, CA 93012, USA) for 24 h and 48 h. The zone of inhibition (ZOI) was measured and the results were compared.

#### 4.3.10. Statistical Analysis 

The study was performed in triplicate and the results are shown as mean ±SD. The statistical analysis was performed by using one-way ANOVA between the samples. The study was performed using Graph Pad Instat (Graph Pad Software Inc., La Jolla, CA, USA).

## Figures and Tables

**Table 1:** Composition of butenafine bilosomes with vesicle size (Y_1_) and entrapment efficiency (Y_2_).

Formulation Code	Independent Constraints			Dependent Constraints	
	Lipid (%)	Surfactant (mg)	Bile Salt (mg)	Vesicle Size (nm)	Entrapment Efficiency (%)
	A	B	C	Y_1_	Y_2_
F1	1.5	30	20	181.5 ± 4.5	67.79 ± 1.5
F2	4.5	30	20	310.1 ± 7.4	85.99 ± 1.8
F3	1.5	60	20	144.4 ± 4.8	76.88 ± 1.4
F4	4.5	60	20	222.8 ± 5.2	91.29 ± 2.1
F5	1.5	45	15	180.8 ± 3.5	69.87 ± 1.3
F6	4.5	45	15	262.7 ± 6.2	87.56 ± 1.8
F7	1.5	45	25	168.9 ± 4.1	72.12 ± 1.4
F8	4.5	45	25	276.3 ± 6.3	86.99 ± 1.7
F9	3	30	15	268.2 ± 5.9	77.38 ± 1.4
F10	3	60	15	208.2 ± 4.2	80.99 ± 1.6
F11	3	30	25	258.8 ± 5.7	75.46 ± 1.4
F12	3	60	25	195.3 ± 4.5	86.50 ± 1.7
F13 *	3	45	20	209.6 ± 4.7	80.23 ± 2.7
F14 *	3	45	20	208.1 ± 4.4	80.98 ± 2.1
F15 *	3	45	20	209.3 ± 3.5	80.12 ± 1.4
F16 *	3	45	20	209.9 ± 4.8	80.21 ± 1.9
F17 *	3	45	20	207.9 ± 5.1	80.56 ± 1.3

* Center point (common composition); values are expressed as mean ± SD, *n* = 3.

**Table 2:** Regression values of responses after applying different experimental models.

Model	Y₁ (Vesicle Size, nm)				
	SD	R²	Adjusted R²	Predicted R²	*p*-Value
Linear	12.85	0.9275	0.9108	0.8625	<0.0001
2FI	11.54	0.9551	0.9281	0.8283	0.1717
Quadratic	5.36	0.9932	0.9845	0.9328	0.0029
	Y₂ (Entrapment efficiency, %)				
Linear	1.37	0.9634	0.9549	0.9242	<0.0001
2FI	0.70	0.9926	0.9881	0.9654	0.0009
Quadratic	0.38	0.9985	0.9966	0.9876	0.0077

**Table 3:** ANOVA test for the quadratic model for vesicle size (Y_1_) and entrapment efficiency (Y_2_).

Source	Particle Size (nm)				Entrapment Efficiency (%)			
	Sum of the Square	DF	F-Value	*p*-Value	Sum of the Square	DF	F-Value	*p*-Value
Model	29,427.84	9	114.00	<0.0001 *	663.11	9	521.14	<0.0001 *
A	19,700.13	1	686.83	<0.0001 *	530.89	1	3755.08	<0.0001 *
B	7727.73	1	269.42	<0.0001 *	105.42	1	745.62	<0.0001 *
C	52.69	1	1.84	0.2174 **	3.47	1	24.56	0.0016 *
AB	651.02	1	22.70	0.0020 *	3.59	1	25.40	0.0015 *
AC	162.31	1	5.66	0.0490 *	1.99	1	14.06	0.0072 *
BC	3.12	1	0.11	0.7514 **	13.80	1	97.62	<0.0001 *
A^2^	49.05	1	1.71	0.2323 **	0.29	1	2.08	0.1929 **
B^2^	206.58	1	7.20	0.0314 *	1.97	1	13.91	0.0074 *
C^2^	855.84	1	29.84	0.0009 *	1.88	1	13.33	0.0082 *
Residual	200.78	7		<0.0001 *	0.99	7		
Lack of Fit	116.15	3	1.83	<0.0001 *	0.46	3	1.18	0.4221 **
Pure Error	84.63	4			0.52	4		
Total	29,628.62	16			664.10	16		

* Significant α < 0.05, ** nonsignificant α > 0.05.

**Table 4:** HET-CAM irritation score of the BN-BSog, sodium lauryl sulfate (2%, positive control), and normal saline (0.9% negative control).

Samples	Egg	Score														
		Nonirritant				Mild Irritant			Moderate Irritant			Severe Irritant				Overall Score
		Time (min)				Time (min)			Time (min)			Time (min)				
		0	0.5	2	5	0.5	2	5	0.5	2	5	0	0.5	2	5	
BN-BSog	Egg 1	0	0	0	0.0	0	0	0	0	0	0	0	0	0	0	0
	Egg 2	0	0	0	0.0	0	0	0	0	0	0	0	0	0	0	
	Egg 3	0	0	0	0.2	0	0	0	0	0	0	0	0	0	0	
	Mean score	0	0	0	0.2	0	0	0	0	0	0	0	0	0	0	
Positive control (SLS, 2%)	Egg 1	0	0	0	0	0	0	0	0	0	0	0	14	17	19	17.44
	Egg 2	0	0	0	0	0	0	0	0	0	0	0	16	18	19	
	Egg 3	0	0	0	0	0	0	0	0	0	0	0	16	18	20	
	Mean score	0	0	0	0.	0	0	0	0	0	0	0	15.33	17.66	19.33	
Negative control (NS, 0.9%)	Egg 1	0	0	0	0	0	0	0	0	0	0	0	0	0	0	0
	Egg 2	0	0	0	0	0	0	0	0	0	0	0	0	0	0	
	Egg 3	0	0	0	0	0	0	0	0	0	0	0	0	0	0	
	Mean score	0	0	0	0	0	0	0	0	0	0	0	0	0	0	

**Table 5:** Levels of independent constraints used in butenafine bilosomes.

Independent Variables	Dependent Variables	
	Lower (−1)	Upper (+1)
Lipid (%)	1.5	4.5
Surfactant (Span 60, mg)	30	60
Bile salt (mg)	15	25

## Data Availability

Not applicable.

## References

[1] Mendes I.T., Ruela A.L.M., Carvalho F.C., Freitas J.T.J., Bonfilio R., Pereira G.R. (2019). Development and characterization of nanostructured lipid carrier-based gels for the transdermal delivery of donepezil. Colloids Surf. B Biointerfaces.

[2] Al-Mahallawi A.M., Abdelbary A.A., Aburahma M.H. (2015). Investigating the potential of employing bilosomes as a novel vesicular carrier for transdermal delivery of tenoxicam. Int. J. Pharm..

[3] Singh D., Pradhan M., Nag M., Singh M.R. (2015). Vesicular system: Versatile carrier for transdermal delivery of bioactives. Artif. Cells Nanomed. Biotechnol..

[4] Ahmed S., Kassem M.A., Sayed S. (2020). Bilosomes as Promising Nanovesicular Carriers for Improved Transdermal Delivery: Construction, in vitro Optimization, ex vivo Permeation and in vivo Evaluation. Int. J. Nanomed..

[5] Niu X.Q., Zhang D.P., Bian Q., Feng X.F., Li H., Rao Y.F., Shen Y.M., Geng F.N., Yuan A.R., Ying X.Y. (2019). Mechanism investigation of ethosomes transdermal permeation. Int. J. Pharm. X.

[6] Zhang Y., Jing Q., Hu H., He Z., Wu T., Guo T., Feng N. (2020). Sodium dodecyl sulfate improved stability and transdermal delivery of salidroside-encapsulated niosomes via effects on zeta potential. Int. J. Pharm..

[7] Pandit A.P., Omase S.B., Mute V.M. (2020). A chitosan film containing quercetin-loaded transfersomes for treatment of secondary osteoporosis. Drug Deliv. Transl. Res..

[8] Maione-Silva L., de Castro E.G., Nascimento T.L., Cintra E.R., Moreira L.C., Cintra B.A.S., Valadares M.C., Lima E.M. (2019). Ascorbic acid encapsulated into negatively charged liposomes exhibits increased skin permeation, retention and enhances collagen synthesis by fibroblasts. Sci. Rep..

[9] Zafar A., Alruwaili N.K., Imam S.S., Hadal Alotaibi N., Alharbi K.S., Afzal M., Ali R., Alshehri S., Alzarea S.I., Elmowafy M. (2021). Bioactive Apigenin loaded oral nano bilosomes: Formulation optimization to preclinical assessment. Saudi Pharm. J..

[10] El Menshawe S.F., Aboud H.M., Elkomy M.H., Kharshoum R.M., Abdeltwab A.M. (2020). A novel nanogel loaded with chitosan decorated bilosomes for transdermal delivery of terbutaline sulfate: Artificial neural network optimization, in vitro characterization and in vivo evaluation. Drug Deliv. Transl. Res..

[11] Khalil R.M., Abdelbary A., Kocova El-Arini S., Basha M., El-Hashemy H.A. (2019). Evaluation of bilosomes as nanocarriers for transdermal delivery of tizanidine hydrochloride: In vitro and ex vivo optimization. J. Liposome Res..

[12] Albash R., El-Nabarawi M.A., Refai H., Abdelbary A.A. (2019). Tailoring of PEGylated bilosomes for promoting the transdermal delivery of olmesartan medoxomil: In-vitro characterization, ex-vivo permeation and in-vivo assessment. Int. J. Nanomed..

[13] Fetih G. (2016). Fluconazole-loaded niosomal gels as a topical ocular drug delivery system for corneal fungal infections. J. Drug Deliv. Sci. Technol..

[14] Soliman O.A.E., Mohamed E.A., Khatera N.A.A. (2019). Enhanced ocular bioavailability of fluconazole from niosomal gels and microemulsions: Formulation, optimization, and in vitro-in vivo evaluation. Pharm. Dev. Technol..

[15] Garg A.K., Maddiboyina B., Alqarni M.H.S., Alam A., Aldawsari H.M., Rawat P., Singh S., Kesharwani P. (2021). Solubility enhancement, formulation development and antifungal activity of luliconazole niosomal gel-based system. J. Biomater. Sci. Polym. Ed..

[16] Kumar N., Goindi S. (2021). Development, characterization and preclinical evaluation of nanosized liposomes of itraconazole for topical application: 32 full factorial design to estimate the relationship between formulation components. J. Drug Deliv. Sci. Technol..

[17] Shetty S., Jose J., Kumar L., Charyulu R.N. (2019). Novel ethosomal gel of clove oil for the treatment of cutaneous candidiasis. J. Cosmet. Dermatol..

[18] Rao S., Barot T., Rajesh K.S., Jha L.L. (2016). Formulation, optimization and evaluation of microemulsion based gel of Butenafine Hydrochloride for topical delivery by using simplex lattice mixture design. J. Pharm. Investig..

[19] Ahmed M.M., Fatima F., Anwer M.K., Ibnouf E.O., Kalam M.A., Alshamsan A., Aldawsari M.F., Alalaiwe A., Ansari M.J. (2021). Formulation and in vitro evaluation of topical nanosponge-based gel containing butenafine for the treatment of fungal skin infection. Saudi Pharm. J..

[20] Alshehri S., Imam S.S. (2021). Formulation and evaluation of butenafine loaded PLGA-nanoparticulate laden chitosan nano gel. Drug Deliv..

[21] Mahdi W.A., Bukhari S.I., Imam S.S., Alshehri S., Zafar A., Yasir M. (2021). Formulation and Optimization of Butenafine-Loaded Topical Nano Lipid Carrier-Based Gel: Characterization, Irritation Study, and Anti-Fungal Activity. Pharmaceutics.

[22] Fachel F.N.S., Medeiros-Neves B., Dal Prá M., Schuh R.S., Veras K.S., Bassani V.L., Koester L.S., Henriques A.T., Braganhol E., Teixeira H.F. (2018). Box-Behnken design optimization of mucoadhesive chitosan-coated nanoemulsions for rosmarinic acid nasal delivery-In vitro studies. Carbohydr. Polym..

[23] Virupakshappa P.K., Krishnaswamy M.B., Mishra G., Mehkri M.A. (2016). Optimization of Crude Oil and PAHs Degradation by *Stenotrophomonas rhizophila* KX082814 Strain through Response Surface Methodology Using Box-Behnken Design. Biotechnol. Res. Int..

[24] Jazuli I., Annu Nabi B., Moolakkadath T., Alam T., Baboota S., Ali J. (2019). Optimization of Nanostructured Lipid Carriers of Lurasidone Hydrochloride Using Box-Behnken Design for Brain Targeting: In Vitro and In Vivo Studies. J. Pharm. Sci..

[25] Yusuf M., Sharma V., Pathak K. (2014). Nanovesicles for transdermal delivery of felodipine: Development, characterization, and pharmacokinetics. Int. J. Pharm. Investig..

[26] Yang G., Wu F., Chen M., Jin J., Wang R., Yuan Y. (2019). Formulation design, characterization, and in vitro and in vivo evaluation of nanostructured lipid carriers containing a bile salt for oral delivery of gypenosides. Int. J. Nanomed..

[27] Gonzalez-Rodriguez M.L., Arroyo C.M., Cozar-Bernal M.J., Gonzalez R.P., Leon J.M., Calle M., Canca D., Rabasco A.M. (2016). Deformability properties of timolol-loaded transfersomes based on the extrusion mechanism. Statistical optimization of the process. Drug Dev. Ind. Pharm..

[28] Abdelbary G., El-Gendy N. (2008). Niosome-encapsulated gentamicin for ophthalmic controlled delivery. AAPS PharmSciTech.

[29] Aboud H.M., Ali A.A., El-Menshawe S.F., Elbary A.A. (2016). Nanotransfersomes of carvedilol for intranasal delivery: Formulation, characterization and in vivo evaluation. Drug Deliv..

[30] Talele P., Sahu S., Mishra A.K. (2018). Physicochemical characterization of solid lipid nanoparticles comprised of glycerol monostearate and bile salts. Colloids Surf. B Biointerfaces.

[31] Mosallam S., Sheta N.M., Elshafeey A.H., Abdelbary A.A. (2021). Fabrication of Highly Deformable Bilosomes for Enhancing the Topical Delivery of Terconazole: In Vitro Characterization, Microbiological Evaluation, and In Vivo Skin Deposition Study. AAPS PharmSciTech.

[32] Uprit S., Kumar Sahu R., Roy A., Pare A. (2019). Preparation and characterization of minoxidil loaded nanostructured lipid carrier gel for effective treatment of alopecia. Saudi Pharm. J..

[33] Fasolo D., Pippi B., Meirelles G., Zorzi G., Fuentefria A.M., Poser G.V., Teixeira H.F. (2020). Topical delivery of antifungal Brazilian red propolis benzophenones-rich extract by means of cationic lipid nanoemulsions optimized by means of Box-Behnken Design. J. Drug Deliv. Sci. Technol..

[34] Zhang W., Zhao X., Yu G., Suo M. (2021). Optimization of propofol loaded niosomal gel for transdermal delivery. J. Biomater. Sci. Polym. Ed..

[35] Higuchi T. (1963). Mechanism of sustained- action medication. Theoretical analysis of rate of release of solid drugs dispersed in solid matrices. J. Pharm. Sci..

[36] Ansari M., Kazemipour M., Aklamli M. (2006). The study of drug permeation through natural membranes. Int. J. Pharm..

[37] Haigh J.M., Smith E.W. (1994). The selection and use of natural and synthetic membranes for in vitro diffusion experiments. Eur. J. Pharm. Sci..

[38] Vinardell M., Mitjans M. (2008). Alternative methods for eye and skin irritation tests: An overview. J. Pharm. Sci..

